# Surface Engineering and Design Strategy for Surface‐Amorphized TiO_2_@Graphene Hybrids for High Power Li‐Ion Battery Electrodes

**DOI:** 10.1002/advs.201500027

**Published:** 2015-05-26

**Authors:** Tengfei Zhou, Yang Zheng, Hong Gao, Shudi Min, Sean Li, Hua Kun Liu, Zaiping Guo

**Affiliations:** ^1^ Institute for Superconducting and Electronic MaterialsAustralian Institute for Innovative Materials (AIIM)School of Mechanical, Materials and Mechatronics EngineeringUniversity of WollongongNorth WollongongNSW2500Australia; ^2^ School of Materials Science and EngineeringUniversity of New South WalesNSW2052Australia

**Keywords:** energy storage, lithium‐ion batteries, low‐temperature phase transformations, surface engineering, titanium dioxide

## Abstract

**Surface amorphization** provides unprecedented opportunities for altering and tuning material properties. Surface‐amorphized TiO_2_@graphene synthesized using a designed low temperature‐phase transformation technique exhibits significantly improved rate capability compared to well‐crystallized TiO_2_@graphene and bare TiO_2_ electrodes. These improvements facilitates lithium‐ion transport in both insertion and extraction processes and enhance electrolyte absorption capability.

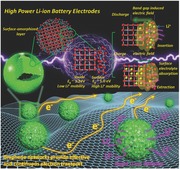

Electrode materials with battery‐like high capacity and capacitor‐like rate performance are highly desirable, since they would significantly advance next‐generation energy storage technology.^1^ TiO_2_ has received increasing attention as an anode material for lithium‐ion batteries (LIBs) due to its good reversible capacity and low volume expansion upon lithiation, as well as its low cost and safe lithiation potential.^2^ The low lithium‐ion mobility within the crystalline phase TiO_2_, however, together with its poor electrical conductivity, means that only a thin surface layer of the host material is available for Li intercalation at high rates.^3^ These issues are still challenges that hinder the electrochemical performance of this material.

Several strategies have been proposed to address each of the issues mentioned above individually. The low lithium‐ion mobility has been addressed by (i) decreasing the length of the lithium‐ion diffusion pathways, for example, by using mesoporous,^3^ nanostructured, or nanosized materials; or by (ii) facilitating the lithium‐ion mobility, for example, by using less dense structural phases.^4^ On the other hand, the conductivity has been improved by (i) coating TiO_2_ with carbon^5^ or Al_2_O_3_,^6^ or (ii) linking TiO_2_ with carbon nanotubes (CNTs) or graphene or other conductive networks,^7^ or (iii) doping the TiO_2_. Traditionally, electrode materials have been sought that have well‐crystallized phases, so that nearly all high energy‐density cathodes for rechargeable lithium batteries are well‐crystallized materials in which lithium and other cations occupy distinct sites.^8^ This “ordering paradigm” may have led the community to overlook a large class of materials in which there are atomic arrangements with only short‐range order that appear to exist in a random (disordered) fashion; some of these materials may offer higher capacity and better stability relative to the well‐crystallized phases.^9^ Interestingly, very recent work has demonstrated that amorphous/disordered materials may form percolation pathways via the opening of active diffusion channels, which could not only potentially facilitate ionic and electronic diffusion, but also enhance mass transport within these phases.^10^ The Li‐ion diffusion in amorphous materials proceeds more rapidly than in crystalline materials with similar particle size and morphology.^9^, ^11^ For instance, Siegel et al. have shown that the ionic conductivity of amorphous Li_2_O_2_ is 2 × 10^−7^ S cm^−1^, which is 12 orders of magnitude larger than that in the crystalline phase.^12^ In addition, defects induced by amorphous phases, such as dangling bonds and oxygen vacancies, typically lead to unexpected electronic states in the band gap that result in improved electrical conductivity.^13^ Once a certain limit is reached, however, a change in the opposite direction is inevitable. Samuelis et al. found that amorphous‐rich material exceeding certain levels of oxygen vacancies is detrimental to the lithium‐ion diffusivity because they represent defects that hinder the mobility of lithium ions.^14^ Therefore, the approach of introducing amorphous phases requires well‐designed surface microstructures and combination with other strategies to further improve the electrochemical performance.

Fortunately, surface‐amorphization (SA) provides unprecedented opportunities for altering and tuning material properties. SA could facilitate lithium diffusion into the bulk materials and alter the electrochemical reactivity to Li, thus increasing the accessible volume to near‐full capacity. Based on these considerations, we propose a robust hierarchical network architecture with anti‐aggregation properties through the assembly of graphene and SA‐TiO_2_, which provides (i) large surface area and electrolyte absorption capability compared with other nanostructures, leading to adequate electrode–electrolyte contact; (ii) lower Li^+^ interfacial transfer resistance due to close packing of the TiO_2_ and graphene, and (iii) improved electronic conductivity and rapid ion/electron transport pathways induced by the SA, which are of benefit to the high power performance. Inspired by these unique properties of SA‐TiO_2_, we can imagine that the lithium‐ion storage properties of TiO_2_ electrodes could be further optimized by combining a hierarchical network architecture and with the surface amorphization of TiO_2_.

Here, we report a unique SA‐TiO_2_@graphene architecture with a remarkable high rate capability, achieved by coupling the advantages of the highly conductive network offered by graphene with the surface amorphization. The surface amorphization was achieved by a low temperature (65 °C) phase transition using titanium glycolate as precursor. The low temperature drives the titanium glycolate to transform into anatase phase and allows a surface‐amorphized layer to be generated under mild conditions during the phase transition. After that, a simple ultraviolet (UV)‐assisted reduction technique was used to construct hybrid architectures combining SA‐TiO_2_ with graphene as complementary building blocks. The resulting SA‐TiO_2_@graphene architecture offers the unique characteristics needed for high‐power anode materials. As a consequence, the optimal SA‐TiO_2_@graphene hybrid architecture exhibits substantial improvements in both cycling stability and rate capability. The composite electrode exhibits a high specific capacity of 103 mA h g^−1^ at a rate of 50 C, 9.3 times higher than that of the reference TiO_2_. It also shows superior cycling capacity (≈108 mA h g^−1^) over 1500 cycles at the high rate of 20 C, together with ≈100% Coulombic efficiency, proving its excellent tolerance of ultrafast insertion and extraction of lithium ions for long‐life LIBs.

In order to prepare surface‐amorphized TiO_2_, a low temperature phase transformation method was used in our work, because it offers the possibility of better controlling the reaction pathways on the molecular level.^15^ It has been reported that the synthesis of TiO_2_ by conventional sol–gel methods is difficult to achieve, as the hydrolysis rates of the precursors are too fast, which, in turn, does not allow separation between the nucleation and growth stages. In order to overcome this problem, ethylene glycol (EG) was selected as a solvent as well as a reactant. Glycols are attractive, as when mixed with alkoxide, they are reactive enough to form glycolate precursors or mixed alkoxide/glycolate derivatives displaying much lower hydrolysis rates. They not only act as an oxygen‐supplying agent for the metal oxide, but also strongly influence surface, particle size, morphology, and assembly properties. The slow hydrolysis rates, mainly a consequence of the moderate reactivity of the C–O bond, in combination with the stabilizing effect of the organic species, lead to the formation of low‐crystallinity products that are often characterized by uniform particle morphologies and tunable crystallite sizes in the range of just a few nano­meters.^15^ Inspired by these advantages, uniform titanium glyco­late spheres were prepared and chosen as precursor, because materials with a spherical morphology have high packing density and good particle mobility to form a compact electrode layer, and actually exhibit the optimal material morphology in conventional electrode fabrication.^16^ Our investigation of the synthetic processes indicated that the reaction temperature was the most important factor, because it critically influences the crystallization and the surface microstructure of the product. If the temperature was too low (room temperature), no anatase phase or crystalline product could be obtained. If the reaction temperature was too high (above 120 °C), however, the rate of reaction and the crystallinity of the product were high, and there was no surface‐amorphized layer generated under these conditions. During the phase transformation, the density difference between titanium glycolate and TiO_2_ results in cracks on the sphere's surface. Since the titanium glycolate spheres have low density, and the spheres are quite loose, H_2_O can penetrate through the cracks on the surface and enter the inner part of the titanium glycolate sphere (**Figure** **1**a). Thus, hydrolysis also occurs within the precursor spheres and leads to the formation of small TiO_2_ particles in the surface layer. These small TiO_2_ particles could act as nuclei for further growth. As the phase transformation proceeds, the TiO_2_ particles grow gradually into interconnected nanoparticles.^17^ We found that at relatively low temperatures (65 °C), the surfaces of the interconnected nanoparticles was constructed from even smaller primary TiO_2_ particles with only short‐range order and unbonded atoms. The hierarchical nanoporous structure of TiO_2_ with a surface‐amorphized layer is thus formed under mild conditions during the phase transformation.

**Figure 1:**
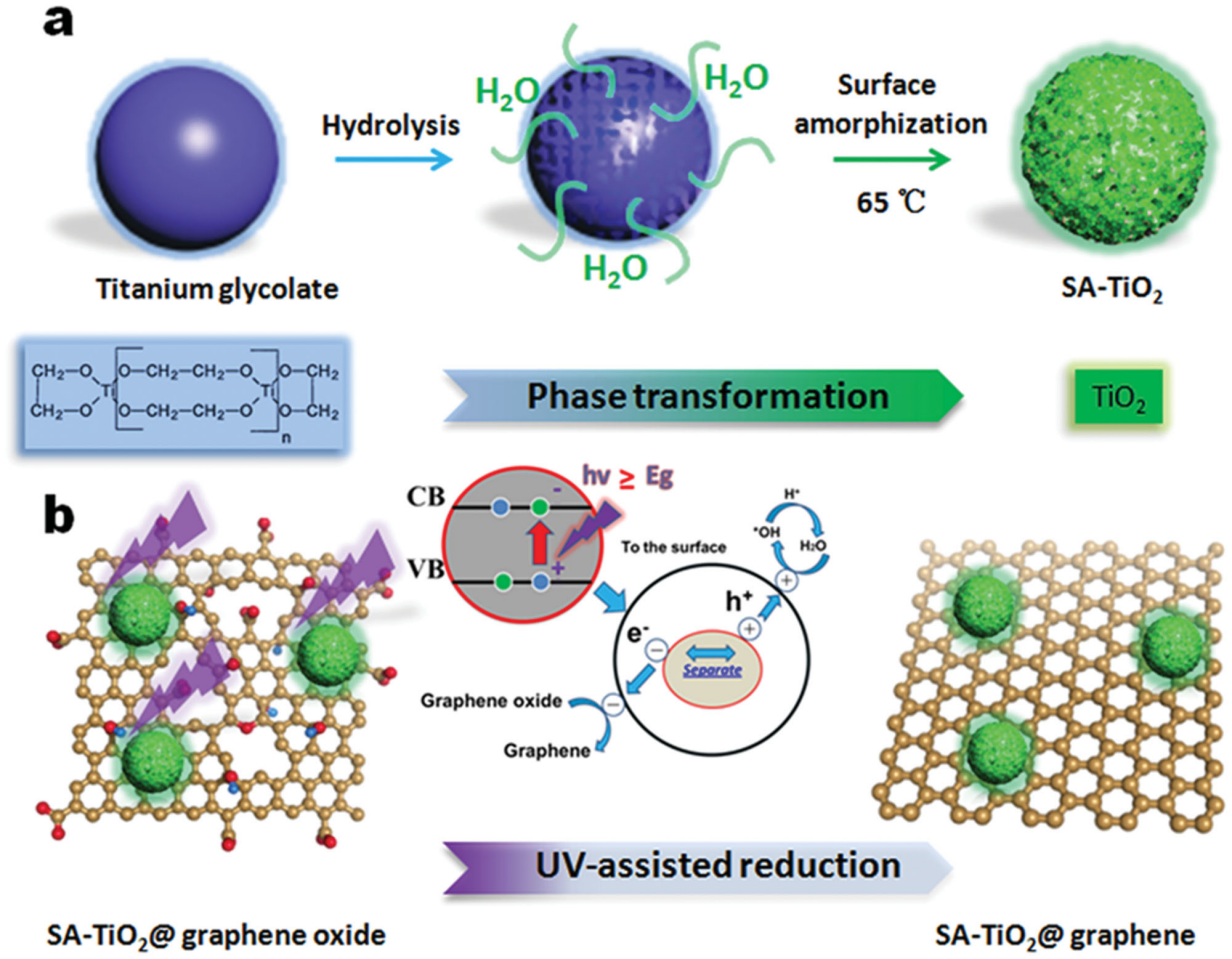
Schematic illustration of the preparation of SA‐TiO_2_@graphene in two steps. a) Phase transformation between titanium glycolate and TiO_2_ results in nanoporous structure on the sphere's surface; the hierarchical nanoporous structure of TiO_2_ with a surface‐amorphized layer is thus formed under low temperature during the phase transformation at a low hydrolysis rate. b) The UV‐assisted reduction technique was used to construct hybrid architectures combining SA‐TiO_2_ with graphene as complementary building blocks.

After successful preparation of the SA‐TiO_2_, we mixed it with graphene oxide (GO) in ethanol and subjected it to steady state UV irradiation. The total amount of GO used for assembly with TiO_2_ was 2.0 wt%. A change in color from ivory white to dark brown can be observed as the reduction of GO proceeds (Figure S1, Supporting Information). This color change has previously been suggested as reflecting partial restoration of the network within the carbon structure and has been witnessed through chemical reduction of the GO sheets. Since TiO_2_ particles carry surface charge in suspensions, they can be readily suspended in polar solvents. Thus, the SA‐TiO_2_‐GO composite becomes readily soluble in polar solvents. Upon UV irradiation of a de‐aerated suspension of SA‐TiO_2_, one observes charge separation. In the presence of ethanol, the holes are scavenged to produce ethoxy radicals, thus leaving the electrons to accumulate within the TiO_2_ particles, as described in Reaction (1).^18^ The accumulated electrons then interact with the graphene oxide sheets and reduce the amounts of certain functional groups [Reaction (2)]. In the present experiments, graphene oxide, with its extended epoxy and carboxylate groups, readily interacts with TiO_2_ particles and undergoes reduction under UV irradiation. The surface hydroxyl groups of TiO_2_ undergo charge transfer interaction with carboxylic acid functional groups on GO, and thus, the direct interaction between SA‐TiO_2_ and graphene provides the basis to obtain SA‐TiO_2_@graphene composite

(1) $${\mathrm{TiO}}_{2}+C_{2}H_{5}\mathrm{OH}\to {\mathrm{TiO}}_{2}(e^{-}+h^{+})\overset{hv}{\to }{\mathrm{TiO}}_{2}(e^{-}){+}^{•}C_{2}H_{4}\mathrm{OH}+H^{+}$$

(2) $${\mathrm{TiO}}_{2}(e^{-})+\mathrm{graphene}\;\;\mathrm{oxide}\;(\mathrm{GO})\overset{hv}{\to }{\mathrm{TiO}}_{2}+\mathrm{graphene}$$

The morphology and microstructure of the SA‐TiO_2_ on graphene sheets were investigated by transmission electron microscopy (TEM) and scanning electron microscopy (SEM). It is interesting to see from the TEM images (**Figure** **2**a,b) that the nanospheres consist of closely interconnected nanoparticles forming a mesoporous network of TiO_2_, where the uniform TiO_2_ nanospheres have a size of ≈100 nm. This sort of porous structure could also offer excellent capability to accommodate strain and structural changes during battery cycling. The corresponding selected area electron diffraction (SAED) pattern (inset image in Figure 2b) shows clear diffraction rings, and it, along with the EDS spectrum (Figure S2, Supporting Information) recorded from the corresponding area, suggests the polycrystalline nature of the whole nanosphere, and matches well with tetragonal anatase. A high resolution TEM (HRTEM) image confirms the crystalline features of the closely interconnected TiO_2_ nanoparticles inside the nanospheres by clear lattice fringes (Figure 2c, Figure S3, Supporting Information). The lattice fringes with a spacing of 3.5 Å can be assigned to the most stable and frequently observed anatase TiO_2_ (101) planes, although it is interesting to find from the HRTEM image (Figure 2c) that the outer part of the nanoparticles consists of surface‐amorphized structures forming an amorphous shell. The magnified TEM image shows that the thickness of the amorphous shell in Figure 2e,g is about 3.0 nm. TEM image of individual sphere (Figure S3a, Supporting Information) shows the SA‐TiO_2_ nanoparticles were composed of numerous primary nanocrystals with sizes in the range of 5–6 nm. Due to the low crystallization temperature and nucleation rate, great deals of primary nanocrystals with poor crystallinity were produced firstly in the reaction solution. Moreover, these tiny TiO_2_ nanocrystals have a large surface to volume ratio and high surface energy, which make them unstable and tend to spontaneous aggregation into larger spherical particles in terms of minimization of the total interfacial energy. A careful view of the individual nanosphere indicates that these tiny nanoparticles loosely contacted with each other, resulting in lots of small pores/channels between them, which was consist with the results of nitrogen isotherm adsorption–desorption curves, the N_2_ hysteresis loop demonstrating the presence of mesopores formed by aggregation of primary particles and the pore size distribution (average pore size ≈7 nm). Compared to the C‐TiO_2_ solid sphere consisted of closely packed particles with size about 50 nm, the much smaller particle size and a large number of mesopores/channels should lead to high surface areas for the SA‐TiO_2_. In addition, more gaseous molecules could be adsorbed on the oxygen vacancies/Ti^3+^ after polarization with an end‐on bound structure and thus lead to high adsorption capacity and then high surface area.[[qv: 10b]] The structure diagram from the simulation of the corresponding surface‐amorphized TiO_2_ shows that the amorphous shell could be constructed of TiO_2_ particles with only short‐range order and unbonded atoms, and these disordered structures not only provide more entrances for Li ions, but also offer plentiful surface oxygen vacancies, significantly increasing the specific surface area and assisting the adsorption capability of the electrolyte (Figure 2f). A thick and milky diffraction background can also be observed in the SAED pattern beside the TiO_2_ diffraction rings, which suggests that the SA‐TiO_2_ contains amorphous or disordered phases, consistent with the HRTEM observations. It is well‐known that graphene fabricated by the UV‐assisted technique has restored *sp* ^2^ networks and multiple available oxygen‐centered surface groups, which will allow unified connection between the GO and the exposed oxygen atoms at the surface of the TiO_2_ nanoparticles.^18^ The SEM images in Figure 2d also clearly show that the uniform TiO_2_ nanospheres with a size of ≈100 nm are well encapsulated by the graphene sheets.

**Figure 2:**
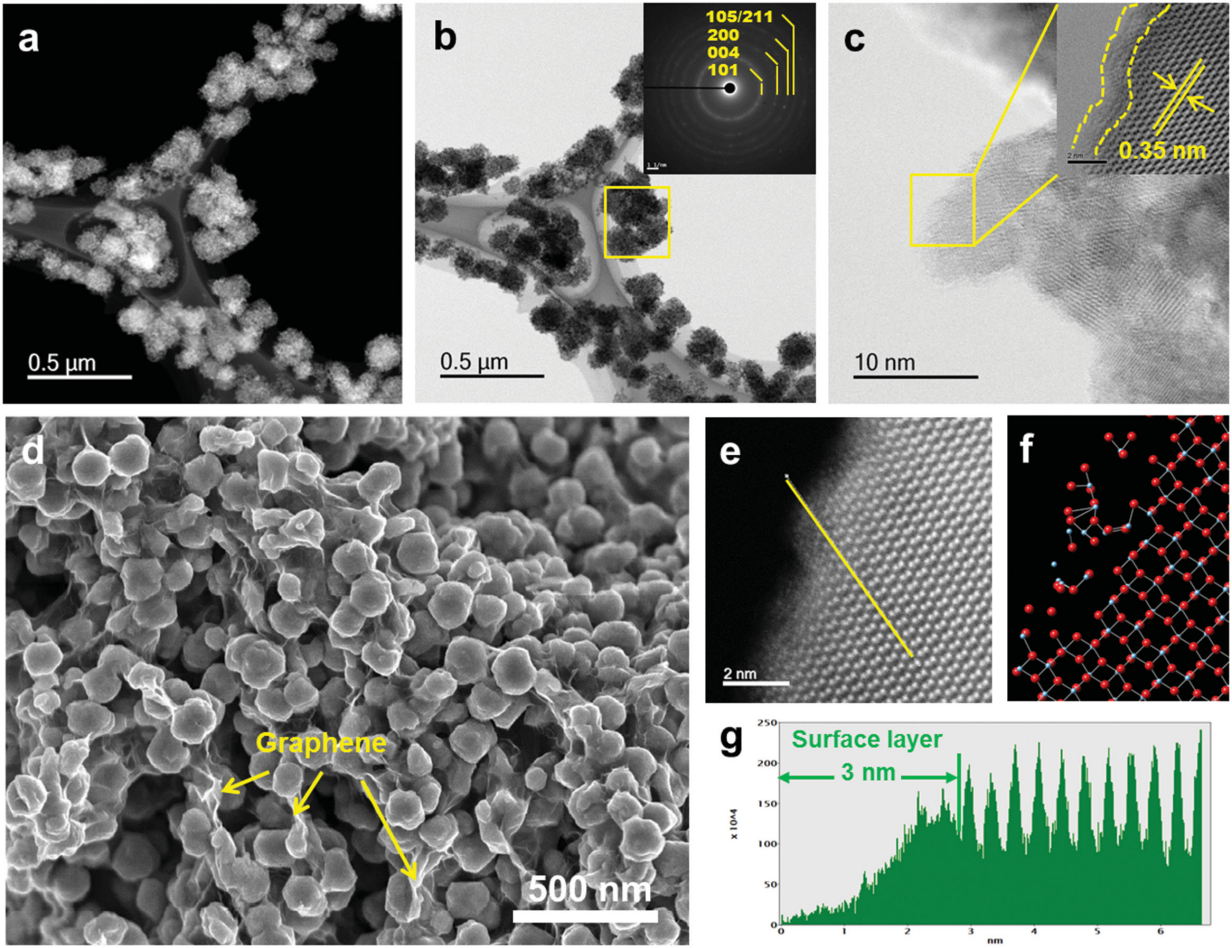
Electron microscope characterization of the SA‐TiO_2_ and SA‐TiO_2_@graphene samples: a) scanning transmission electron microscopy–high angle annular dark‐field imaging (STEM‐HAADF) and b) bright field TEM images of SA‐TiO_2_ with uniform sphere‐like structure (with the inset in panel (b) the SAED pattern of the corresponding area). c) HRTEM image of the surface of a single SA‐TiO_2_ nanosphere, with the inset a magnified view of the region enclosed by the yellow box, which shows a clearly amorphous surface. d) SEM image of SA‐TiO_2_@graphene shows that the uniform TiO_2_ nanospheres are well encapsulated by the graphene. e) Magnified STEM‐HAADF image of the amorphous surface and f) Structure diagram from a simulation of the surface constructed from short‐range‐ordered TiO_2_ particles and unbonded atoms. g) Measurement of surface layer depth corresponding to the line in panel (e).

The crystal structure and microstructure of the samples were further analyzed by X‐ray diffraction (XRD). **Figure** **3**a compares the XRD patterns of SA‐TiO_2_ and SA‐TiO_2_@graphene. All samples show diffraction peaks at 2*θ* = 25.36°, 37.90°, 48.15°, 54.05°, and 62.86°, which can be indexed to the (004), (200), (105), (211), and (204) planes of anatase TiO_2_ (space group: I4 1/amd, JCPDS No. 21–1272), respectively, confirming that the synthesized TiO_2_ is anatase. Besides the distinctive diffraction peaks of anatase TiO_2_, no obvious diffraction peak of graphene is observed for the SA‐TiO_2_@graphene sample, suggesting that the stacking of the graphene sheets remains disordered. We further conducted Raman measurements (Figure S4, Supporting Information). Two obvious carbon bands, the D band and G band peaks, could be observed for both samples. Compared to the crystallized‐TiO_2_@graphene (C‐TiO_2_@graphene) samples, a peak at around 148.7 cm^−1^, corresponding to the characteristic vibrational modes of anatase with a large featureless background, was observed for the SA‐TiO_2_@graphene samples, with the large background in the Raman spectra suggesting that a thin amorphous phase/layer possibly exists on the surface of the SA‐TiO_2_@graphene. Nitrogen isotherm adsorption–desorption curves together, with the pore size distribution for the SA‐TiO_2_@graphene, are presented in Figure 3b. For SA‐TiO_2_@graphene, a type IV isotherm is observed, which is characteristic of mesoporous materials. Based on the Barrett–Joyner–Halenda (BJH) equation, the main pore size (inset in Figure 3b) in SA‐TiO_2_@graphene is 8 nm. The specific surface area of SA‐TiO_2_@graphene is 326 m^2^ g^−1^.

**Figure 3:**
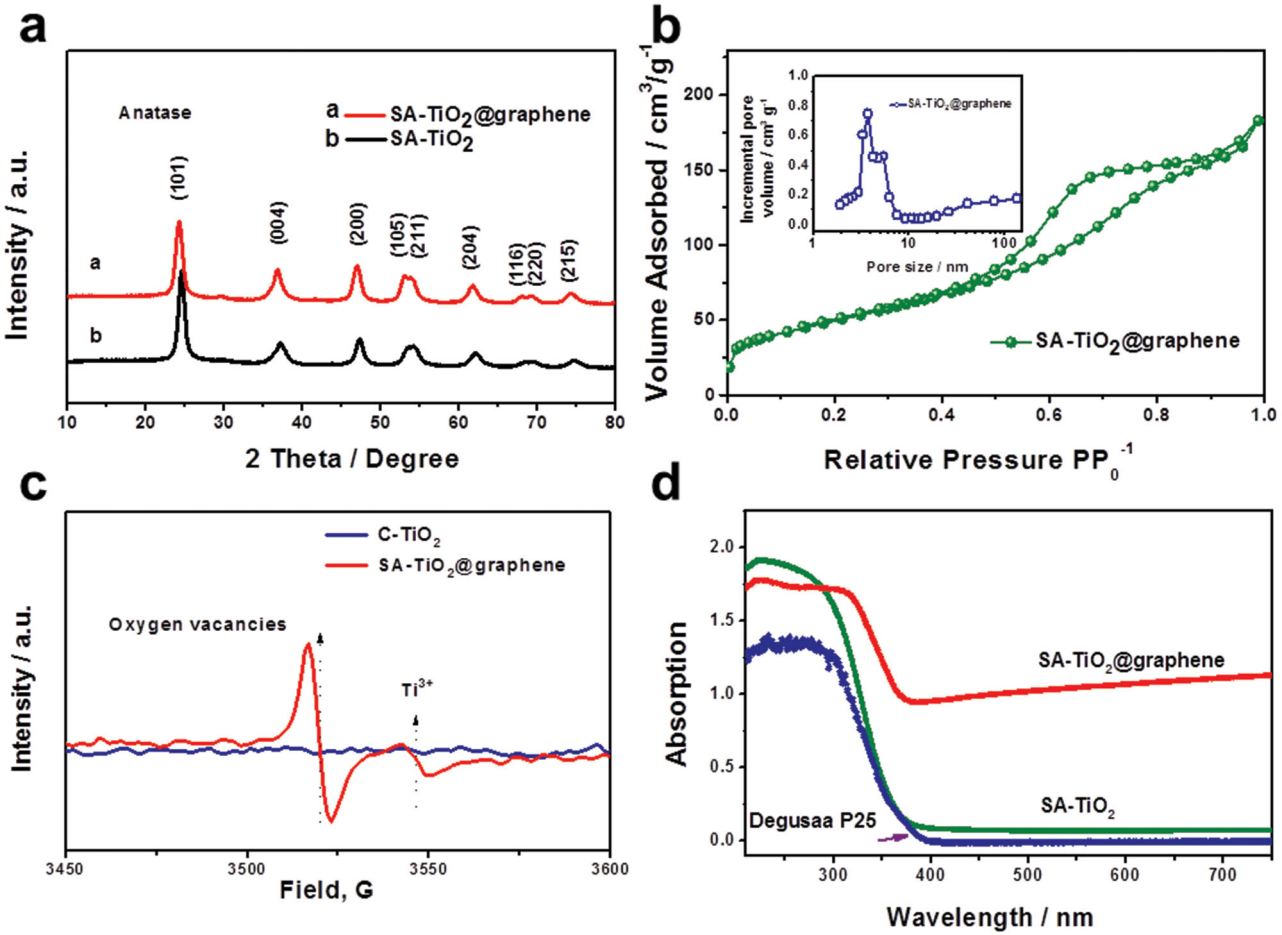
XRD patterns of a) SA‐TiO_2_@graphene and b) SA‐TiO_2_. b) Nitrogen adsorption and desorption isotherms (inset: pore size distribution) of SA‐TiO_2_@graphene. c) EPR spectra for SA‐TiO_2_@graphene and C‐TiO_2_ (C‐TiO_2_ heated in air, details in the Supporting Information). d) UV–vis diffuse reflectance spectra of SA‐TiO_2_@graphene, SA‐TiO_2_, and commercial Degussa P25 TiO_2_ as reference.

In order to further confirm the existence of the amorphous surface layer of TiO_2_ in the samples, electron paramagnetic resonance (EPR) measurements and X‐ray photoelectron spectroscopy (XPS) were conducted to investigate the surface and physical properties of TiO_2_. Compared to the heat‐treated (600 °C in argon) C‐TiO_2_ samples, the appearance of an intense signal for the SA‐TiO_2_@graphene can be ascribed to oxygen vacancies, which may due to the single electron trapped on the oxygen vacancy states in the EPR spectra (Figure 3c). Notably, the creation of surface‐amorphized TiO_2_ not only results in oxygen vacancy formation, but also creates Ti^3+^ interstitials in the TiO_2_ matrix. In both cases, Ti is in excess with respect to O, and they both decrease the optical band gap of TiO_2_. Moreover, oxygen vacancies are not only the most important defects in TiO_2_, but also the most prevalent defects in many oxides, which greatly affect their chemical and physical properties. We have also studied the surface chemical bonding and valence bands of the samples with XPS. The binding energies from the samples were calibrated with respect to the C 1s peak from the carbon tape at 284.8 eV. Ti 2p and O 1s XPS peaks confirmed the formation of TiO_2_ (Figure S5, Supporting Information). The oxygen vacancies close to the surface lead to shifting of the core level of O 1s and the valence band to higher binding energies. The broad and asymmetric nature of the peak is ascribed to the various coordinations of oxygen in the SA‐TiO_2_. The O 1s XPS peak could be fitted into three peaks. The lowest binding energy peak located at 530.4 eV can be assigned to the lattice oxygen species. The highest binding energy peak at 533.9 eV is attributed to the near‐surface oxygen, such as from surface hydroxylation, adsorbed H_2_O. In addition, a new peak located at 532.2 eV is observed in the SA‐TiO_2_ sample, which can be assigned to oxygen vacancies.^19^ There is no clear evidence, however, for Ti^3+^. This is because Ti^3+^ is susceptible to oxidation by O_2_ in air and cannot survive long.^20^ The oxygen vacancies are more likely to be located in the disordered outer layer, considering that the crystalline phases are almost identical and large structural alteration was observed in this layer. The UV/visible (UV/Vis) spectroscopy of SA‐TiO_2_ and SA‐TiO_2_@graphene demonstrated higher absorbance over the full region (200–800 nm) than for the Degussa P25, and a steep increase in light absorbance is observed in both these two samples at wavelengths below 400 nm, which indicates that the photon trapping capacity of SA‐TiO_2_ was significantly enhanced, so that it maintains a high level of absorbance throughout the entire region. The reason for the color change from white (TiO_2_) to ivory white (SA‐TiO_2_) can be explained as follows: a surface‐engineered amorphous TiO_2_ nanoparticle consists of two phases (a crystalline TiO_2_ nanocrystal as a core inside, and a highly disordered surface layer where dopants [oxygen/Ti^3+^ vacancies] are introduced outside). Although an ensemble of single nanocrystals retains the benefits of crystalline TiO_2_ structures, the introduction of disorder and dopant at their surface would enhance visible and infrared absorption, with the additional benefit of carrier trapping.[[qv: 10c]] UV/Vis spectroscopy was used to confirm the presence of oxygen vacancies. It was reported that the SA‐TiO_2_ spectrum features an increase in absorption between 400 and 800 nm due to free electrons, local Ti^3+^ centers (a band at 620 nm), and oxygen vacancies (bands at 1060, 441, and 486 nm).[[qv: 4a]] From the UV/Vis reflectance spectra of SA‐TiO_2_ (Figure 3d), a significant increase in the absorbance compared to Degussa P25 in the visible light region for SA‐TiO_2_ can be observed, which confirms the presence of oxygen vacancies.

To demonstrate the effectiveness of the surface‐amorphized structure of SA‐TiO_2_@graphene in improving rate‐capacity, the lithium‐insertion/extraction properties of SA‐TiO_2_@graphene as anode were investigated by galvanostatic charge–discharge measurements. **Figure** **4**a shows the discharge (Li^+^ insertion) and charge (Li^+^ extraction) curves of a SA‐TiO_2_@graphene electrode at the rate of 0.5 C (84 mA g^−1^). In a voltage window of 1–3 V, the discharge and charge capacities of the 1st cycle are 332 and 273.4 mA h g^−1^, respectively. Clearly, this electrode shows superior cyclic retention to 100 cycles. Both the SA‐TiO_2_@graphene and the SA‐TiO_2_ are found to be effective in improving Li‐storage, showing a greatly enhanced discharge capacity after 100 cycles (205.1 mA h g^−1^ for SA‐TiO_2_@graphene, and 141.7 mA h g^−1^ for SA‐TiO_2_), while only 61.8 mA h g^−1^ was achieved with C‐TiO_2_. In Figure 4c and Figure S6, Supporting Information, the SA‐TiO_2_@graphene showed the typical electrochemical characteristics of anatase TiO_2_, exhibiting cathodic insertion of lithium at 1.75 V and anodic extraction of lithium at 1.95 V versus Li/Li^+^. The better Li^+^ storage performance of the SA‐TiO_2_@graphene in comparison with that of C‐TiO_2_@graphene can be observed from the cyclic voltammograms (CVs) (Figure S6, Supporting Information). The voltage difference between the anodic and cathodic peaks (Δ*E*_p_) reflects the degree of polarization of the electrode. SA‐TiO_2_@graphene has a much lower Δ*E*_p_ value than C‐TiO_2_@graphene. Therefore, the SA‐TiO_2_@graphene is less polarized and has faster electrochemical reaction kinetics. This result agrees well with the electrochemical performance results shown in Figure 4b. The CV curves of both samples remained almost unchanged after three cycles, demonstrating that the electrochemical reaction was stable and reversible. More strikingly, with respect to the reference TiO_2_, the specific capacity of SA‐TiO_2_@graphene is substantially increased at all investigated charge–discharge rates, as demonstrated in Figure 4b,d. Figure 4b and Figure S7, Supporting Information, illustrate the specific charge obtained at different C‐rates (0.5, 1, 2, 5, 10, 20, and 50 C, equal to 0.084, 0.168, 0.336, 0.840, 1.680, 3.360, and 8.400 A g^−1^, respectively) as a function of the number of cycles. For example, the specific capacity of SA‐TiO_2_@graphene at the rate of 50 C is as high as 103 mA h g^−1^, 9.3 times higher than that of the reference C‐TiO_2_. Importantly, after the high rate measurements, the specific capacity of SA‐TiO_2_@graphene at 0.5 C can recover to the initial value, indicating its high reversibility. It also shows superior cycling capacity (≈108 mA h g^−1^) over 1500 cycles at the high rate of 20 C, along with ≈100% Coulombic efficiency, proving its excellent tolerance of ultrafast insertion and extraction of lithium ions for long‐life LIBs.

**Figure 4:**
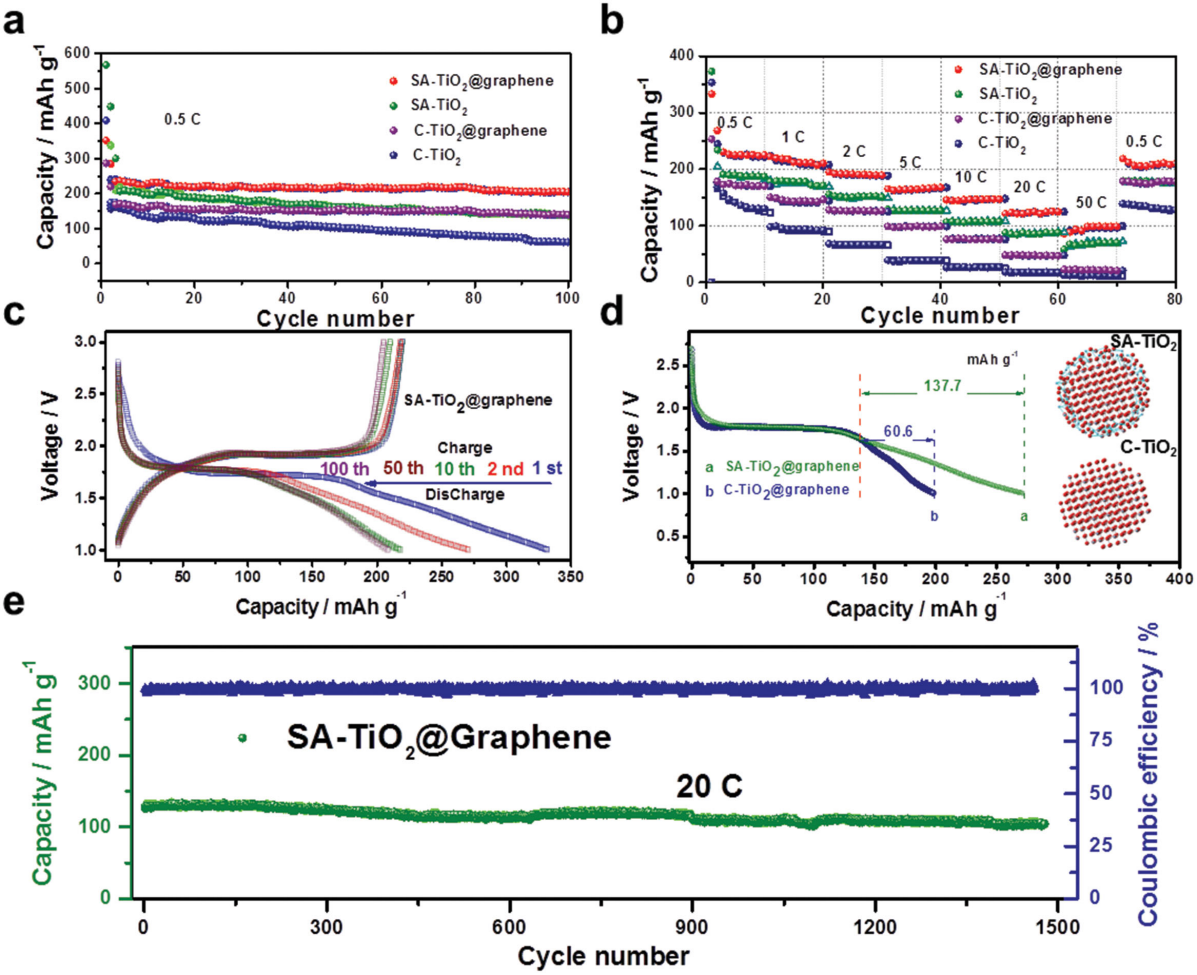
Electrochemical performance of SA‐TiO_2_@graphene electrodes. a) Capacity retention through 100 cycles at 0.5 C for the samples. b) Cycling performance of as‐prepared samples at various current rates. c) Galvanostatic charge/discharge profiles at various cycles. d) Potential profiles of the SA‐TiO_2_@graphene and C‐TiO_2_@graphene for the cathodic cycle at the current density of 0.5 C, with the inset showing the structures of the TiO_2_ particles; e) Long‐term cycling performance of SA‐TiO_2_@graphene at the high current density of 20 C, showing the reversible capacity value of 108 mA h g^−1^ after 1500 cycles with Coulombic efficiency of ≈100%.

In a control experiment, C‐TiO_2_@graphene was synthesized following the same procedures as for the synthesis of the SA‐TiO_2_@graphene and then heat‐treated in air at 600 °C to eliminate the surface‐amorphized layer. The capacity for C‐TiO_2_@graphene is 139.7 mA h g^−1^ (Figure 4a), which is lower than that of the SA‐TiO_2_@graphene (205.1 mA h g^−1^) and higher than that of C‐TiO_2_ (61.8 mA h g^−1^). That is because the particle size and the surface‐amorphized layer of TiO_2_ are of great importance for its electrochemical performance. The distance from the surface to the core drastically affects the ability to store charge at high C‐rates. Because the surface atomic arrangement affects the specific charge at moderate C‐rates, for example, from 0.5 to 50 C, the effective diffusion length for lithium ions is 24 nm under the charge/discharge rate of 0.5 C, and 3.2 nm under 50 C.^21^ The lithium‐ion mobility also becomes extremely low for lithium‐ion content higher than that of Li_0.5_TiO_2_.[[qv: 4a]] Consequently, storing lithium ions beyond Li_0.5_TiO_2_ can only be achieved near the surface, and thus, higher specific charge capacities are obtained with increasing specific surface area. It has been demonstrated that ordinary crystalline anatase TiO_2_ gives only the orthorhombic Li_0.5_TiO_2_, while Li*_x_*TiO_2_ (*x* > 0.5) with higher lithium storage capacity can indeed be formed in ultrasmall TiO_2_ as a result of the quantum‐size confinement effect.^22^ In our work, the thickness of the surface layer is ≈3 nm. The amorphous‐layer‐induced defects such as dangling bonds and oxygen vacancies typically lead to unexpected electronic states in the band gap that result in improved electrical conductivity (Figure S8, Supporting Information). The calculated band gaps for crystalline TiO_2_ and amorphous TiO_2_ are 3.2 and 1.0 eV, respectively.^13^ The surface amorphous layer in our work is different from an amorphous structure with only poor crystallinity.^23^ The surface amorphous layer with its narrow band gap (≈1.0 eV) has plenty of oxygen vacancies and Ti^3+^ ions, which together with the crystalline structure inside (band gap, *E*_g_ = 3.2 eV) induces an electric field during the charge/discharge process to facilitate deeper charge diffusion within the materials (**Figure** **5**). We believe that the TiO_2_ with different band gaps creates an electric field effect for lithium storage that may occur in the deep region of TiO_2_ and thus increases the capacity to some extent. Below the plateau of phase transformation from tetragonal to orthorhombic (≈1.75 V), a sloping curve appears, in which the capacity comes from the lithium‐ion intercalation beyond Li_0.5_TiO_2_ (Figure 4d). The capacity beyond Li_0.5_TiO_2_ for SA‐TiO_2_@graphene is about 137.7 mA h g^−1^, which is nearly double that of C‐TiO_2_@graphene (60.6 mA h g^−1^). The narrow band gap of the surface amorphous layer also improves the electronic conductivity and rapid ion/electron transport pathways, and it also has benefits for high power performance. Moreover, the amorphous layer with high surface area possesses better adsorption capability for ions and molecules to facilitate Li^+^ transfer across the electrolyte/TiO_2_ interface, due to the higher local concentration. In addition, the weak connections between the SA‐TiO_2_ nanospheres are responsible for the poor cycling and rate performance during lithium insertion/extraction processes. When the SA‐TiO_2_ nanospheres are well encapsulated by the graphene, however, using a simple UV‐assisted reduction technique, the graphene provides an interconnected conductive network which significantly improves the cycling and rate performances. The excellent high‐rate performance is attributed to the substantial decrease in charge‐transfer resistance due to the unique structure of the SA‐TiO_2_@graphene.^24^ The stable cycling of an electrode has strict requirements in terms of structural stability at the particle level, because small changes in particle morphology could cause electrode‐level cracking and failure. After 10 deep cycles at the current density of 336 mA g^−1^, the morphology of the SA‐TiO_2_@graphene was examined with SEM and there is no obvious structural change after cycling. As the SEM micrographs in Figure S10d, Supporting Information, demonstrate, we can see a thin solid electrolyte interphase (SEI) film on the sample surface after 10 cycles. The partially reversible SEI formation might also contribute to the enhanced capacity.

**Figure 5:**
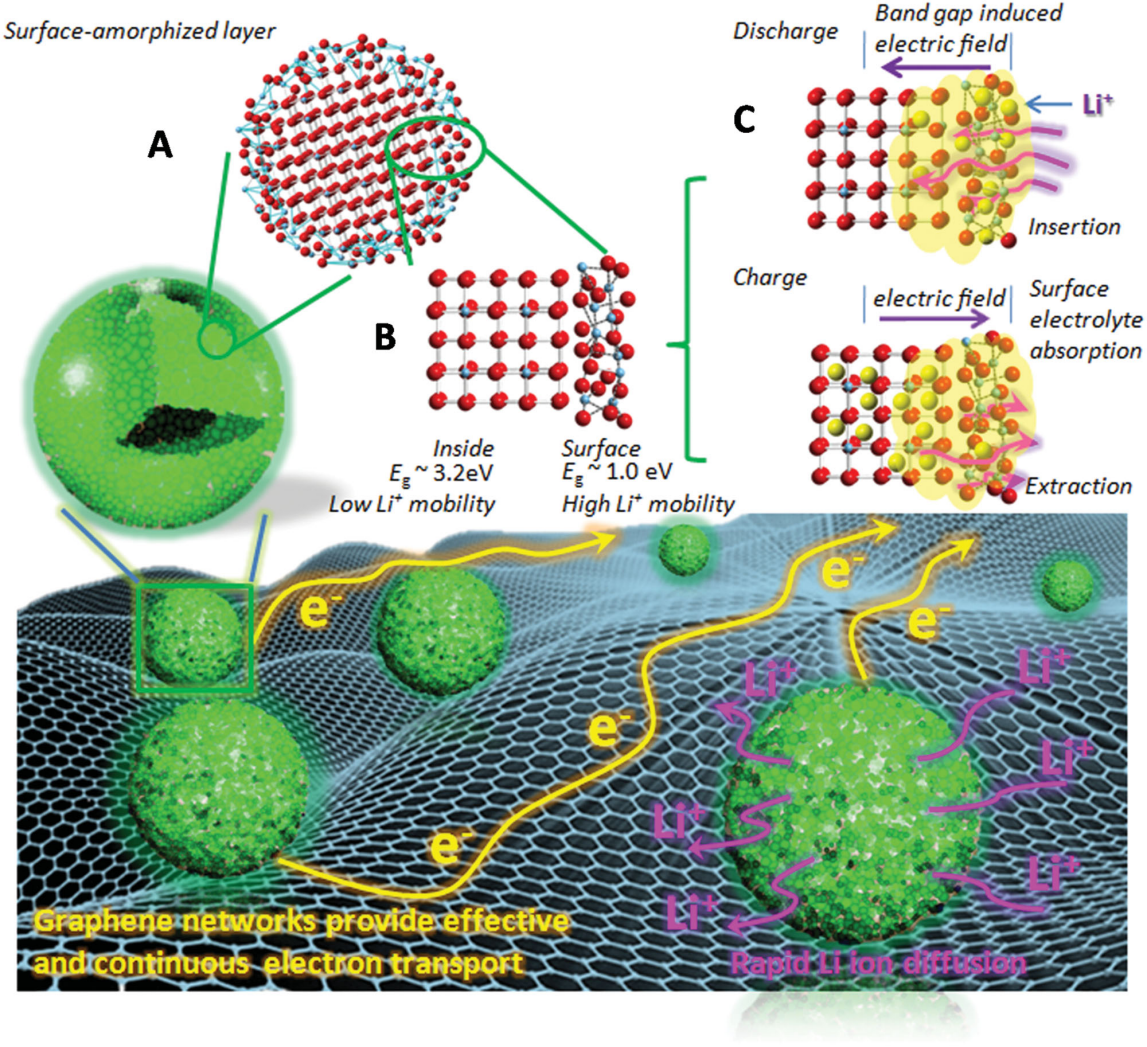
Summary of the enhanced high rate performance mechanism of the SA‐TiO_2_@graphene in the Li battery system. The surface amorphous layer (Region A) with its narrow band gap ≈1.0 eV) has plenty of oxygen vacancies and Ti^3+^ ions, which together with the crystalline structure inside (band gap, *E*_g_ = 3.2 eV) (Region B) induces an electric field during the charge/discharge process to facilitate deeper charge diffusion within the materials (Region C). In the meanwhile, the graphene matrix provides an effective and continuous electrically conducting network. The highly porous surface amorphous layer also helps in electrolyte absorption.

We have designed and successfully fabricated surface amorphized (SA)‐TiO_2_@graphene composites. A thin amorphous TiO_2_ layer was created on crystalline TiO_2_ nanospheres under mild conditions and assembled with graphene via an UV‐assisted reduction technique. We evaluated the SA‐TiO_2_@graphene as an electrode material for lithium‐ion batteries and found that it exhibited greatly improved lithium insertion/extraction performance compared with crystalline TiO_2_. The optimal SA‐TiO_2_@graphene hybrid architecture exhibits a substantial improvement in lithium specific capacity from 0.5 to 50 C. More strikingly, the specific capacity of the composite at the rate of 50 C is as high as 103 mA h g^−1^, 9.3 times higher than that of the reference TiO_2_. We attributed the greatly enhanced lithium storage properties of the SA‐TiO_2_@graphene nanocrystals to the lower lithium‐ion diffusion and electronic conduction resistance, and better surface adsorption in the amorphous layer. These improvements benefit from the electric field introduced by the surface‐amorphization within the nanocrystals, which leads to much lower lithium‐ion diffusion resistance, facilitates its transport in both insertion and extraction processes, and also enhances the electrolyte absorption capability. This concept thus offers an innovative and general approach toward designing battery materials with better performance.

## Supporting information

As a service to our authors and readers, this journal provides supporting information supplied by the authors. Such materials are peer reviewed and may be re‐organized for online delivery, but are not copy‐edited or typeset. Technical support issues arising from supporting information (other than missing files) should be addressed to the authors.

Supplementary
