# Supplementary material for: Surface Engineering and Design Strategy for Surface‐Amorphized TiO2@Graphene Hybrids for High Power Li‐Ion Battery Electrodes
Source: Adv Sci (Weinh). 2015 May 26;2(9):1500027. doi: 10.1002/advs.201500027 (PMC5115387; doi:10.1002/advs.201500027)
Supplement: Supplementary file 1 — Supplementary [file ADVS-2-0r-s001.pdf]

Copyright WILEY-VCH Verlag GmbH & Co. KGaA, 69469 Weinheim, Germany, 2015.

ADVANCED SCIENCE
Open Access

Supporting Information

for *Adv. Sci.*, DOI: 10.1002/advs.201500027

Surface Engineering and Design Strategy for Surface-Amorphized TiO_2@Graphene Hybrids for High Power Li-Ion Battery Electrodes

*Tengfei Zhou, Yang Zheng, Hong Gao, Shudi Min, Sean Li, Hua Kun Liu, and Zaiping Guo**

Copyright WILEY-VCH Verlag GmbH & Co. KGaA, 69469 Weinheim, Germany, 2013.

ADVANCED SCIENCE

Supporting Information

Surface Engineering and Design Strategy for Surface-amorphized TiO_2@Graphene Hybrids towards High Power Li-ion Battery Electrodes

*Tengfei Zhou [1], Yang Zheng [1], Hong Gao [1], Shudi Min [1], Sean Li [2], Hua Kun Liu [1], Zaiping Guo [1] **

T. Zhou, Y. Zheng, H. Gao, S. Min, Prof. H. K. Liu, Prof. Z. Guo
Institute for Superconducting and Electronic Materials, Australian Institute for Innovative Materials (AIIM), School of Mechanical, Materials and Mechatronics Engineering, University of Wollongong, North Wollongong, NSW 2500, Australia
E-mail: zguo@uow.edu.au

Prof. S. Lean
School of Materials Science and Engineering, University of New South Wales, NSW 2052, Australia

Experimental Section

Experimental materials and method for GO sheets

Natural graphite powder was oxidized to graphite oxide by the modified Hummers method. Specifically, graphite powder (0.04 g) was dispersed in 50 ml concentrated H_2SO_4 in an ice bath. 3.0 g $KMnO_4$ was then added gradually. The mixture was stirred for 2 h and then diluted with deionized water. 10 ml of 30% H_2O_2 was then added to the solution until the color of the mixture changed to bright yellow. The graphite oxide (GO) prepared in this way was re-dispersed in deionized water and then exfoliated to GO sheets by ultrasonication. A brown homogeneous supernatant was obtained by repeated centrifuging and washing. Deionized (DI) ultrapure water (18 MΩ. cm^{-1}) was used for solution preparation.

Materials Synthesis and Assembly

Synthesis of Surface-Amorphized TiO_2 Spheres.

2 mL of $Ti(OBu)_4$ was added to 10 mL of ethylene glycol (EG). This mixture was kept under vigorous stirring at room temperature for 12 h. This solution was then quickly poured into 100 mL of acetone. This mixture was kept under vigorous stirring at room temperature for another 2 h, yielding a white precipitate that was harvested by centrifugation. This solid, comprising titanium glycolate spheres, was washed several times with ethanol by successive rounds of centrifugation and removal of the supernatant. In the next step, the titanium glycolate product was added to 20 mL isopropanol and 10 mL H_2O, and this mixture was stirred for 10 h at 65 °C to produce the surface-amorphized-TiO_2 spheres, which were isolated by centrifugation, washed with ethanol several times, and dried in an oven at 60 °C for 24 h.

TiO_2@graphene hybrid particles were prepared via simultaneous photoreduction of GO and TiO_2 precursors. A typical GO reduction was carried out in a 80 mL vial containing an aqueous suspension of surface-amorphized TiO_2 spheres (0.2 g) in the presence of 50 mL ethanol containing 1.0 mL of 4 mg mL-1 GO. The slurry was irradiated in ultraviolet (UV) light ($\lambda \leq 350$ nm) using a 18 W light emitting diode (LED) lamp for 2 h. After irradiation, the

particles were centrifuged and washed with DI water three times. The final products were dried in an oven at 60 °C before use. The reduced GO concentration was calculated to be ~2.0 wt %.

Characterization

The crystalline structure of the as-prepared material was characterized by X-ray diffraction (Rigaku Mini Flex 600). The morphologies and particle sizes of the samples were observed by field emission scanning electron microscopy (FEI-NOVA NanoSEM 450 and Magellan, FEI, USA). The details of the crystal structure were further characterized by transmission electron microscopy (TEM), which was conducted on a JEOL JEM-ARM200F transmission electron microscope operating at 200 kV. Selected area electron diffraction (SAED) patterns were recorded using a Gatan charge coupled device (CCD) camera in a digital format. The TEM was linked to an energy dispersive spectrum analysis (EDS) system. The Brunauer–Emmett–Teller (BET) specific surface areas of the samples were evaluated on the basis of nitrogen adsorption isotherms using a Micromeritics ASAP 2020MC gas adsorption apparatus (USA). X-ray photoelectron spectroscopy (XPS) was conducted on a VG Multilab 2000 (VG Inc.) photoelectron spectrometer using monochromatic Al Kα radiation under vacuum at 2×10^{-6} Pa. All of the binding energies were referenced to the C 1s peak at 284.8 eV of the surface adventitious carbon. The ultraviolet–visible diffuse reflectance spectra (DRS) were measured using the diffuse reflectance method with a Shimadzu UV-2600 spectrophotometer using an integrating sphere accessory. $BaSO_4$ was used as a reflectance standard in ultraviolet–visible diffuse reflectance experiments. Continuous-wave electron paramagnetic resonance (EPR) experiments were carried out using a Bruker ELEXSYS E580 spectrometer operating in the X-band (9.4 GHz) mode and equipped with an Oxford CF935 helium flow cryostat with an ITC-5025 temperature controller. The g tensors were calibrated for homogeneity and accuracy by comparison to a coal standard, $g = 2.00285 \pm 0.00005$. The receiver gain and number of

scans were adjusted to every spectrum of a particular sample to enable comparisons at a reasonable signal-to-noise ratio.

Electrochemical measurements

The electrochemical tests were carried out via CR2032 coin type cells. The working electrodes were prepared by mixing the as-prepared materials, Super P, multi-walled carbon nanotube (MWCNT), and sodium carboxymethyl cellulose / polyacrylic acid (1:1) at a weight ratio of 70:10:10:10. The resultant slurry was pasted on Cu foil and dried in a vacuum oven at 150 °C for 3 h, followed by pressing at 300 kg cm^{-2}. The loading of the materials on individual electrodes was 1.0 ± 0.2 mg cm^{-2}. Electrochemical measurements were carried out using two-electrode coin cells with Li metal as counter and reference electrode. Celgard (product 2400) was used as the separator for the lithium-ion battery. The cell electrolyte for the lithium-ion battery was $LiPF_6$ (1 M) in ethylene carbonate (EC) / diethylene carbonate (DEC) / ethyl-methyl carbonate (EMC) [1:1:1 (v/v/v) with 5 wt% fluoroethylene carbonate (FEC)]. Electrochemical impedance spectroscopy (EIS) and cyclic voltammetry (CV) were conducted on a VMP-3 electrochemical workstation at a scan rate of 0.2 mV s^{-1}. The cells were galvanostatically charged and discharged at different constant current densities, based on the weight of the samples, on a Land CT2001A battery tester. At least five parallel cells were tested for each electrochemical measurement, in order to make sure that the results were reliable and represented the typical behavior of the samples.

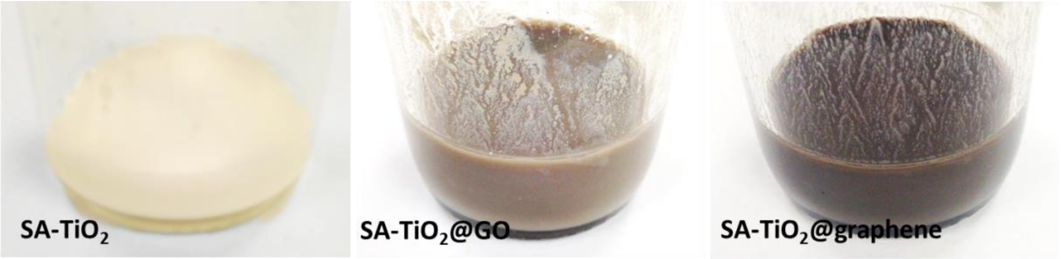

Figure S1. Change in color of SA-TiO_2 nanoparticles with GO before and after UV irradiation for 2 h. A suspension of SA-TiO_2 nanoparticles is also shown for comparison.

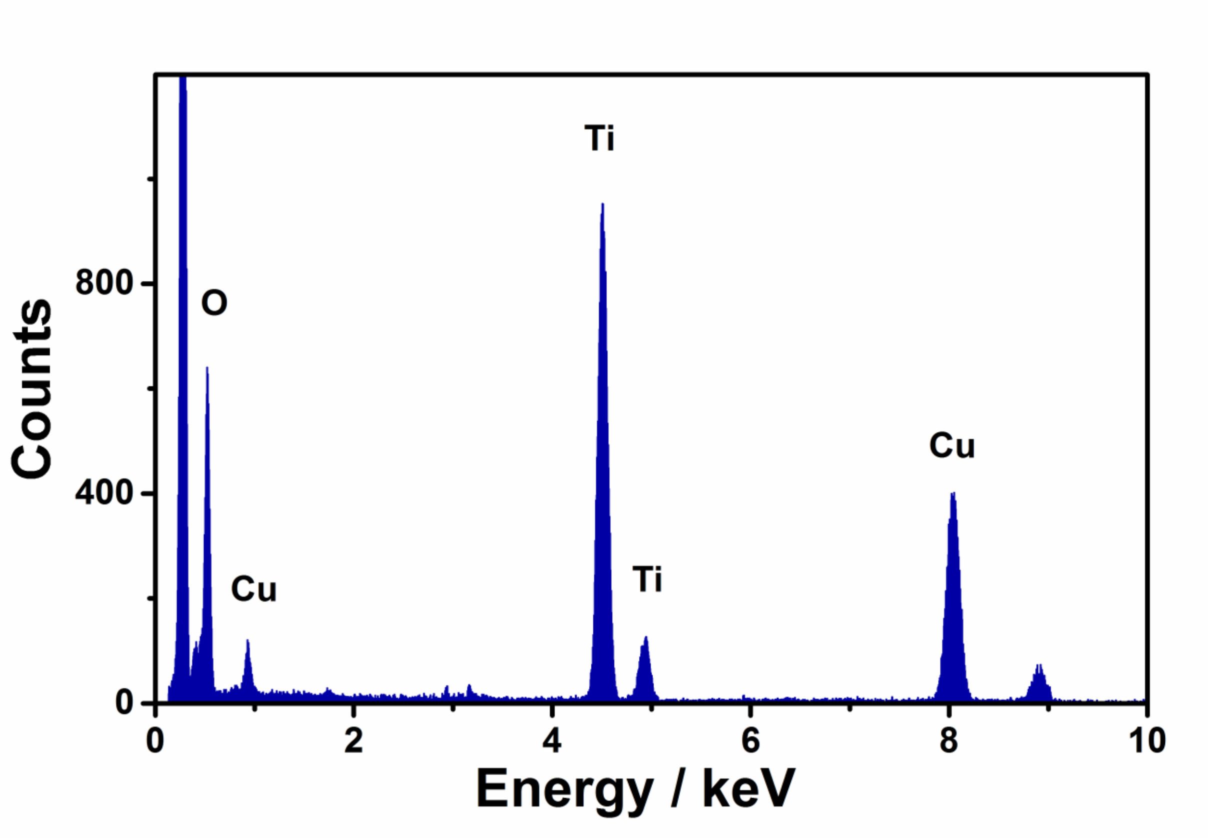

Figure S2. EDS spectrum of SA-TiO_2 nanoparticles.

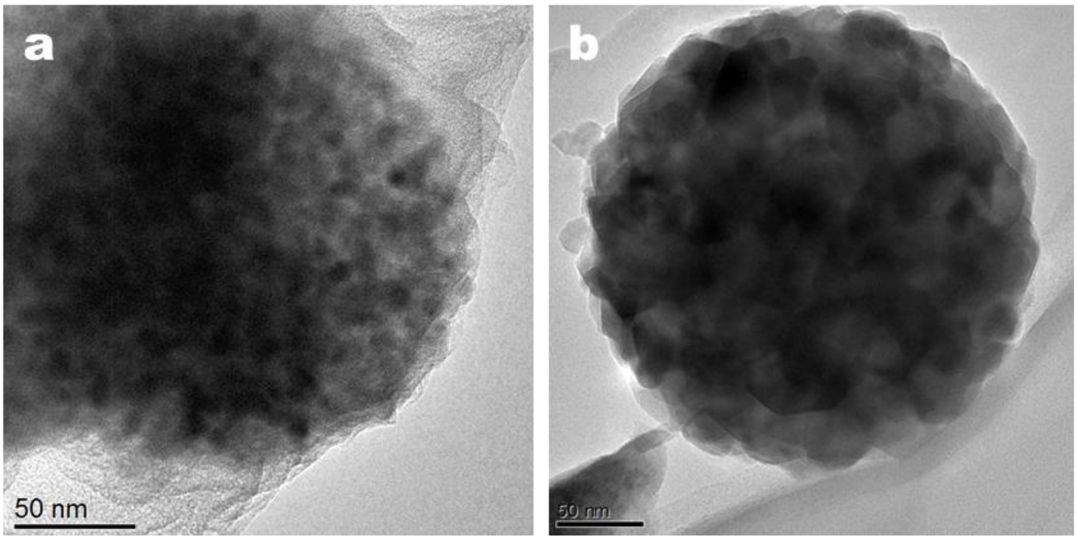

Figure S3. TEM images of SA-TiO_2@graphene (a) and C-TiO_2@graphene (b).

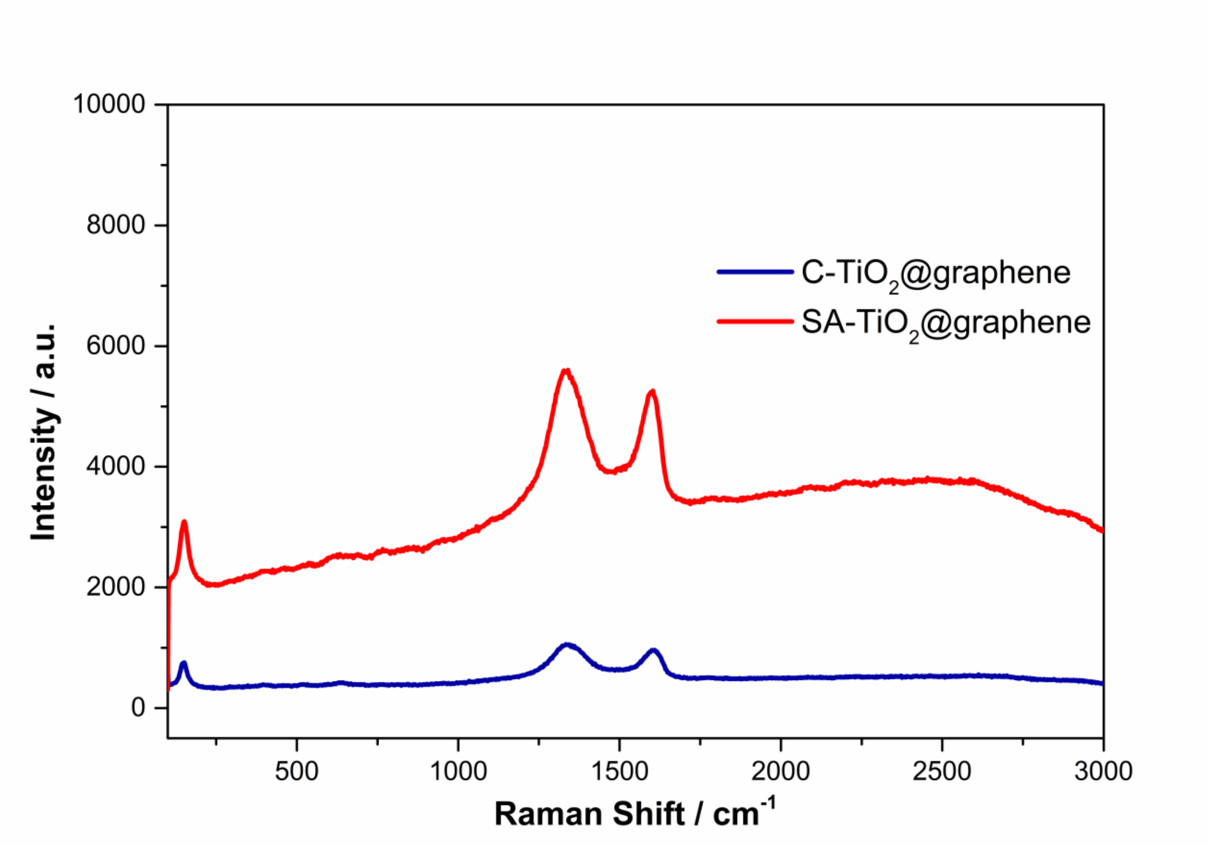

Figure S4. Raman spectra of SA-TiO_2@graphene and C-TiO_2@graphene.

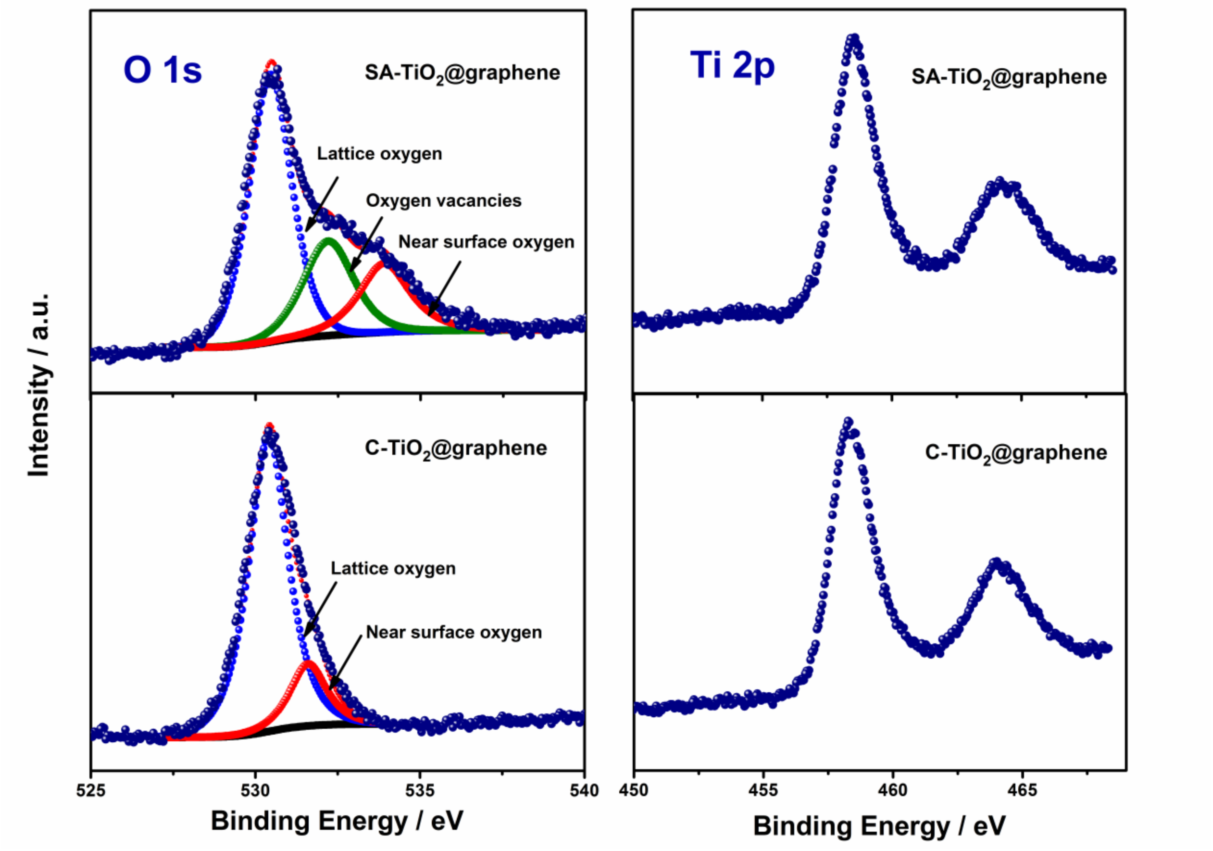

Figure S5. XPS spectra of SA-TiO_2@graphene and C-TiO_2@graphene.

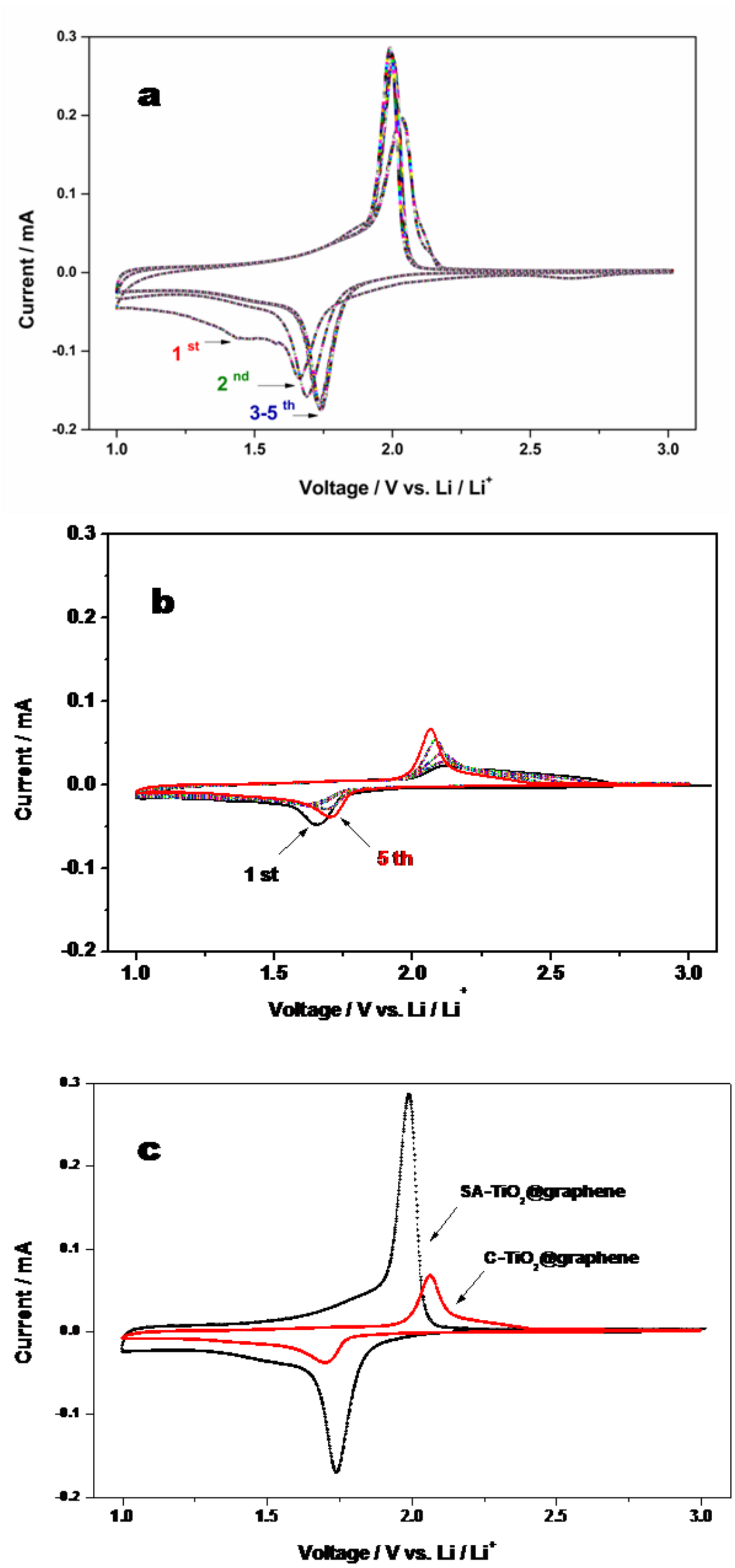

Figure S6. Cyclic voltammograms for the first 5 cycles of (a) SA-TiO_2@graphene, (b) C-TiO_2@graphene; and (c) 5th cycle CVs of both samples for comparison at the scan rate of 0.2 mV/s.

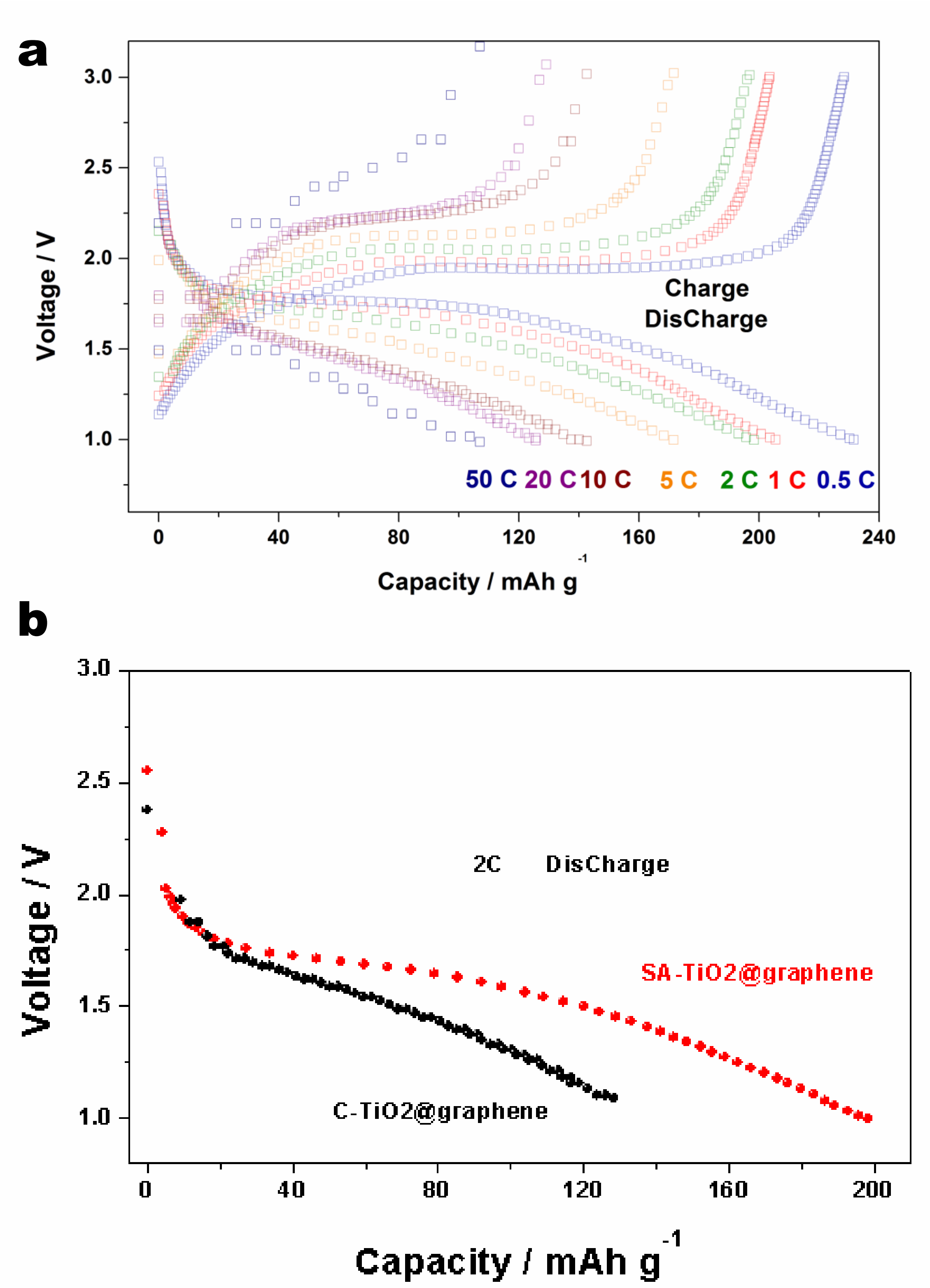

Figure S7. (a) Charge and discharge curves of SA-TiO_2@graphene at different current rates from 0.5 C - 50 C. (b) Potential profiles of the SA-TiO_2@graphene and C-TiO_2@graphene for the cathodic cycle at the current density of 2 C.

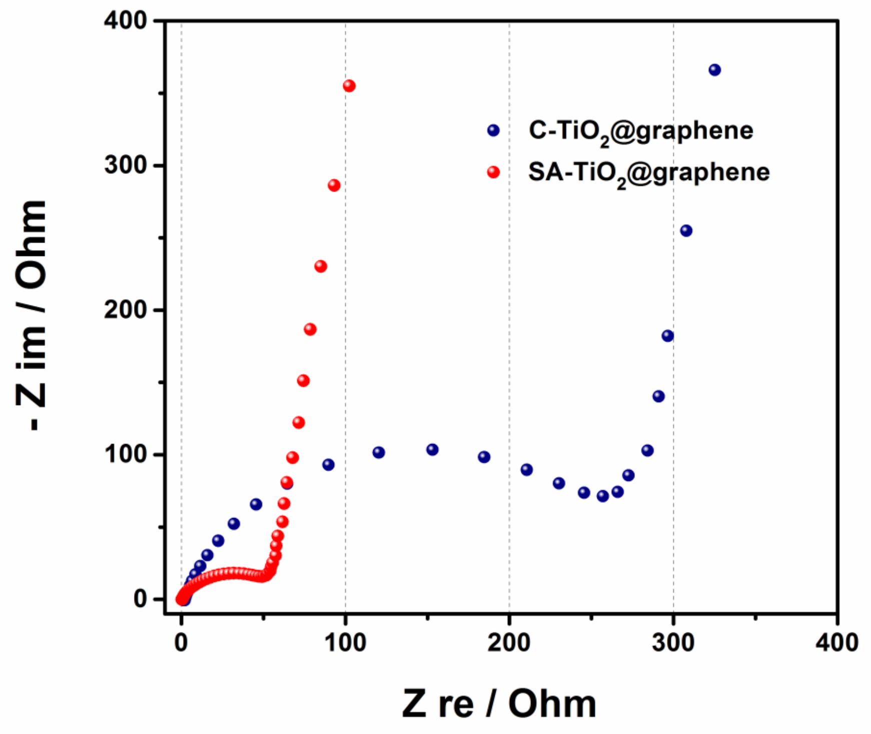

Figure S8. Nyquist plots of electrodes containing hybrid SA-TiO_2@graphene and C-TiO_2@graphene, obtained by applying a sine wave with an amplitude of 5.0 mV over the frequency range from 100 kHz to 0.01 Hz.

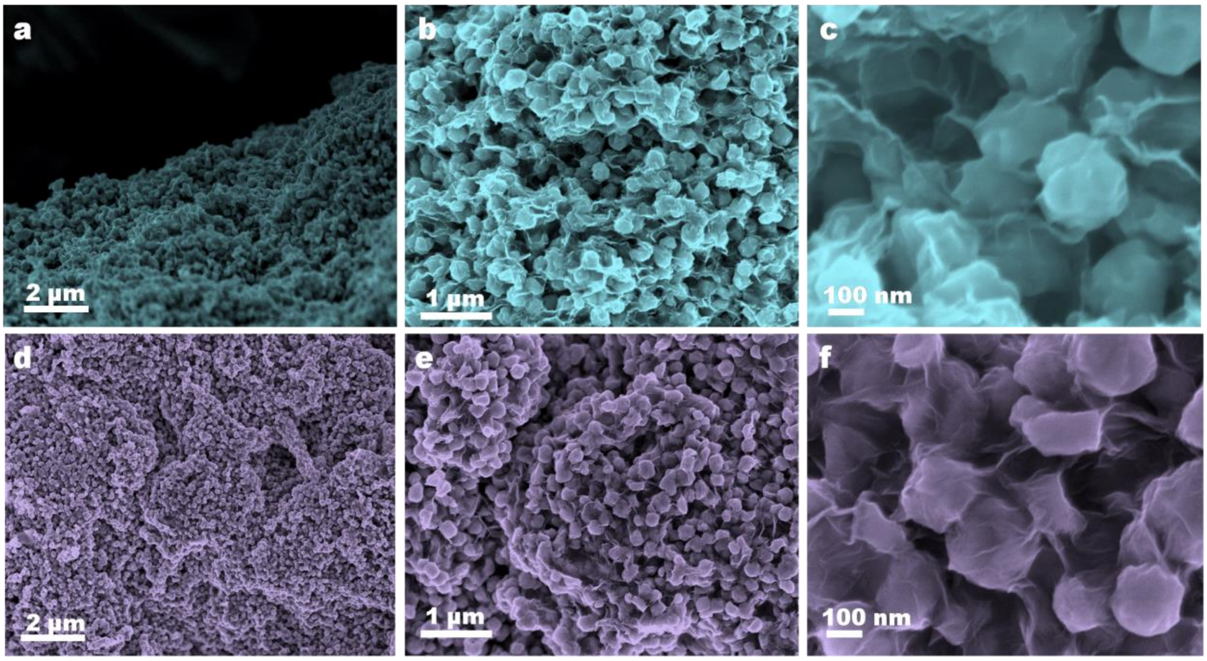

Figure S9. Cross-sectional (a, b, c) and top-view (d,e, f) SEM images of SA-TiO_2@graphene show that the uniform TiO_2 nanospheres are well encapsulated by the graphene.

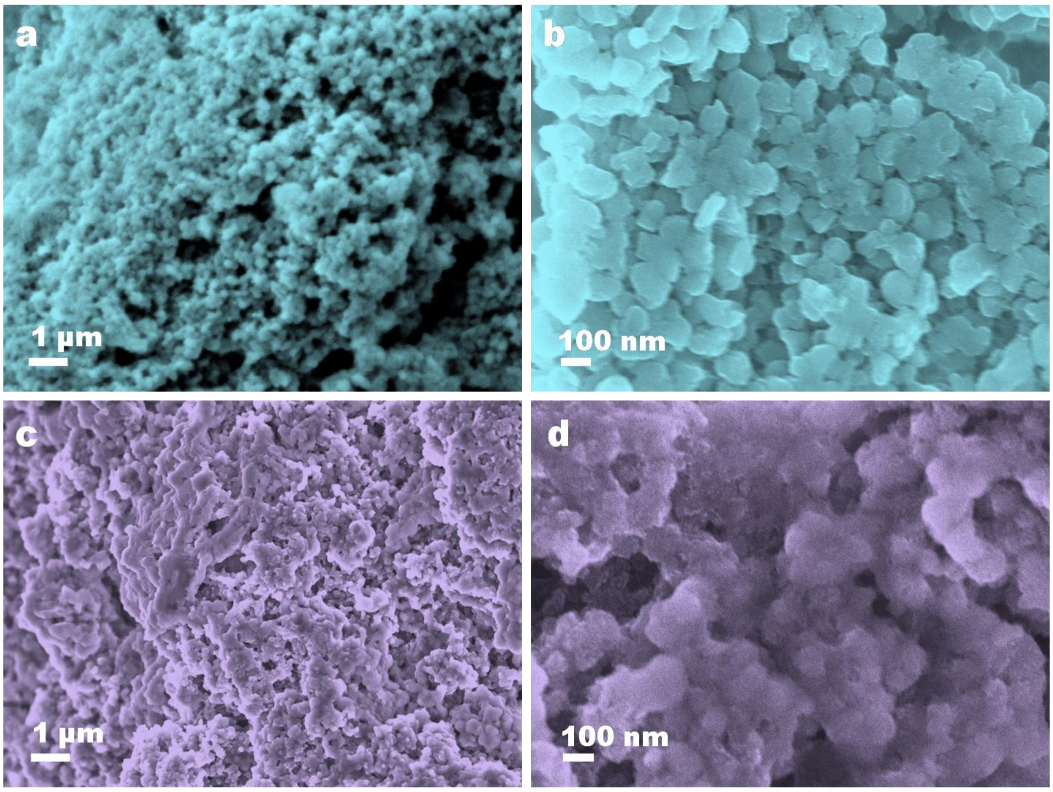

Figure S10. Typical SEM images of SA-TiO_2@graphene electrode before (a,b) and after (c, d) 10 cycles at the current density of 336 mA g^{-1}.

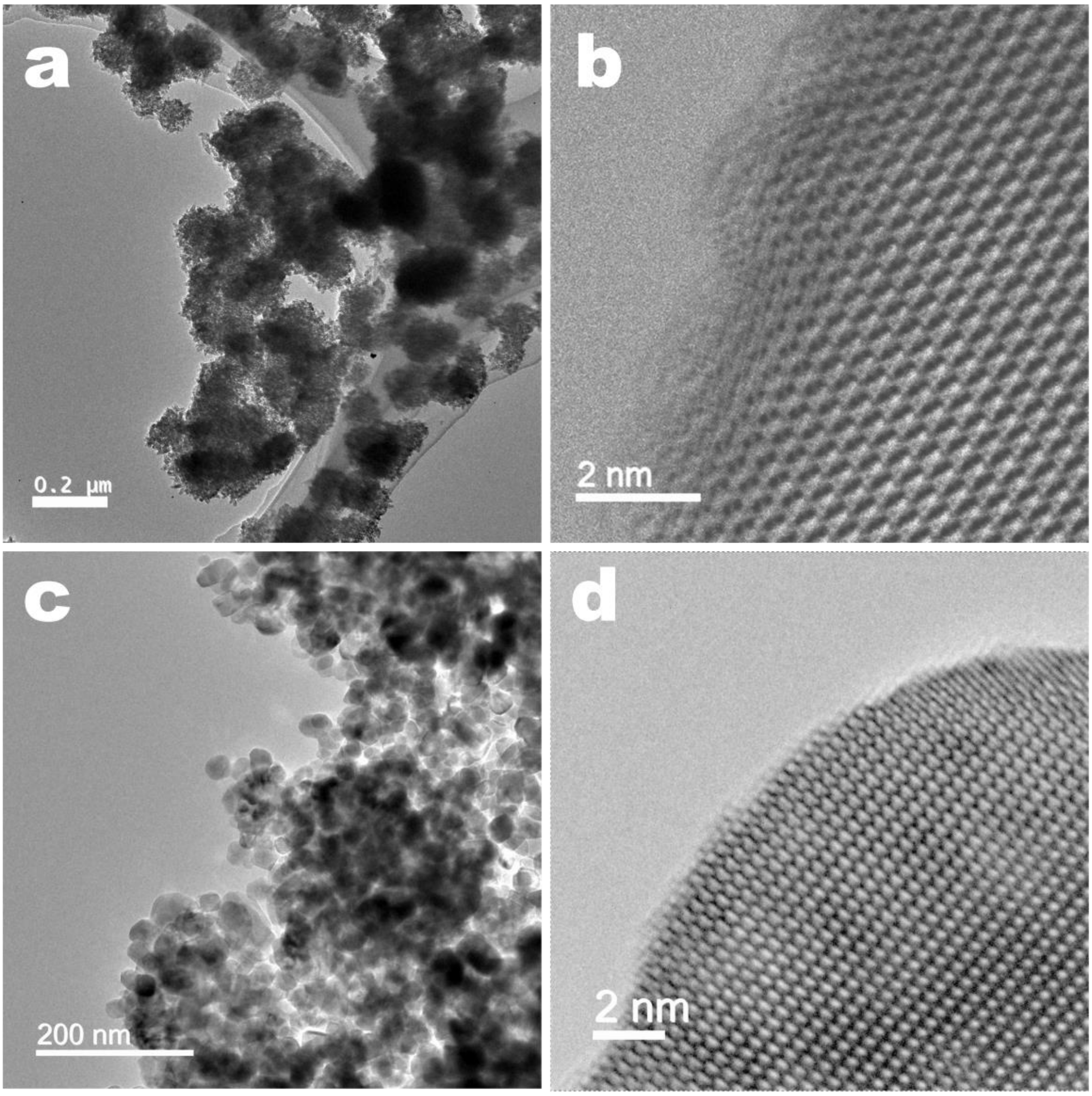

Figure S11. TEM and HRTEM images of SA-TiO_2 (a, b) and C-TiO_2 (c, d), respectively.

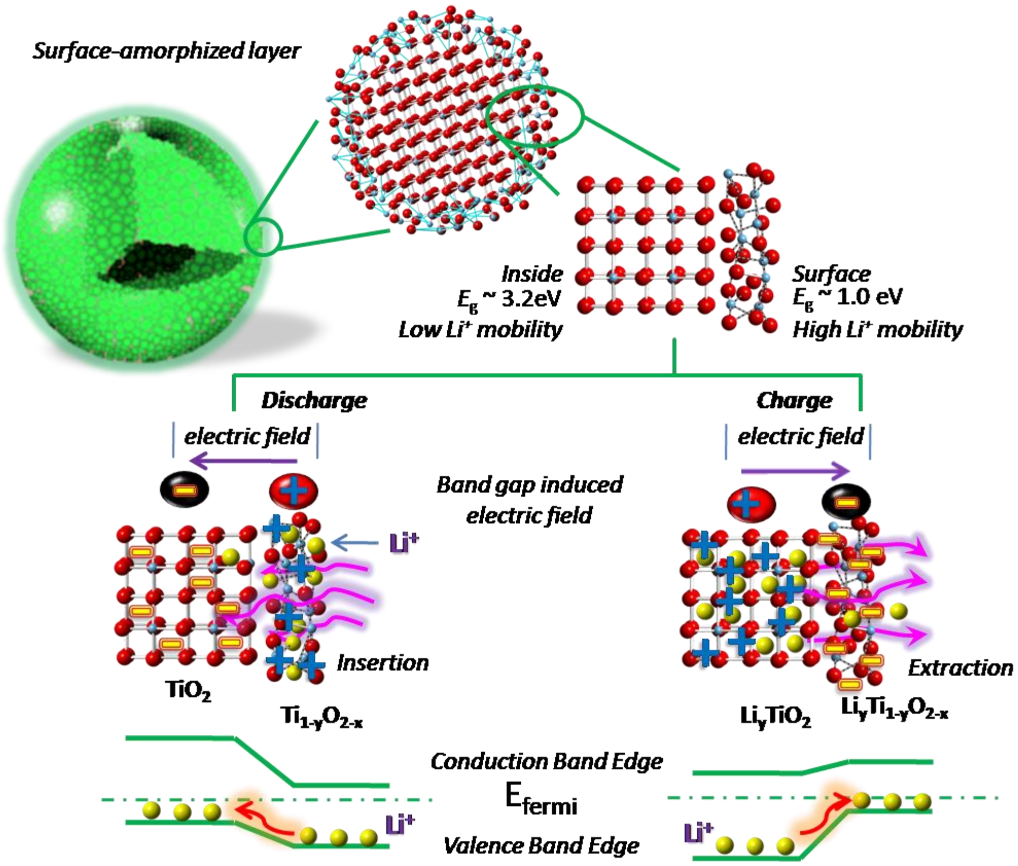

Figure S12. Summary of the enhanced high rate performance mechanism of the SA-TiO_2@graphene in the Li battery system.
